# Microwave Synthesis Under Solvent-Free Conditions and Spectral Studies of Some Mesoporphyrinic Complexes

**DOI:** 10.3390/molecules17055592

**Published:** 2012-05-10

**Authors:** Rica Boscencu

**Affiliations:** Department of Inorganic Chemistry, Faculty of Pharmacy, “Carol Davila” University of Medicine and Pharmacy, 6 Traian Vuia St., 020956 Bucharest, Romania; Email: rboscencu@yahoo.com; Tel./Fax: +4-021-311-1152

**Keywords:** solvent-free synthesis, mesoporphyrinic complexes, microwave assisted synthesis, solvatochromism

## Abstract

A series of A_3_B and A_4_ type mesoporphyrinic complexes were synthesized with superior yields using microwave irradiation under solvent-free conditions. The structures of the complexes were confirmed using elemental analysis, FT-IR, UV-Vis, EPR and NMR spectral data. The influence of environmental polarity on spectral properties of the mesoporphyrinic complexes was investigated. The obtained results indicate that the shape of absorption and fluorescence spectra does not depend on the solvent polarity under the experimental conditions used. The small shifts of the absorption and emission maximums that occur by increasing of solvent polarity reflects the physical interaction between the porphyrinic substituents and the solvent molecules.

## 1. Introduction

The synthesis of mesoporphyrinic complexes and the investigation of their spectral and biological properties have attracted increasing interest in medicinal chemistry, mainly due to their use in photodiagnosis and photodynamic therapy of malignant tumors [1,2,3,4,5,6]. 

Photodynamic therapy involves topical or systemic administration of a pharmaceutical formulation containing photosensitizer, its selective accumulation in the tumor tissue, followed by generation of cytotoxic species such as singlet oxygen, by irradiation with light in the red region of the visible spectrum and subsequent destruction of the sick cells [7,8,9,10]. Photodynamic diagnostics are a cancerous lesion detection procedure based on the accumulation of the photosensitizer at a specific target, which emits fluorescence upon light excitation [3,6,11]. 

The increasing use of metalloporphyrins in diagnosis and therapy is justified by their convenient activation wavelengths, situated in the near-red infrared region, adequate for deep tissue penetration, complemented by their low toxicity and high capacity to generate singlet oxygen [3,4]. A good localization of the metalloporphyrinic compounds at a subcellular level is directly influenced by the amphiphilicity of these structures and plays a major role in their biomedical efficiency [12,13,14]. The degree of purity of the metalloporphyrins used in biomedical purpose is very important because the presence of trace amounts of impurities can introduce significant errors in pharmacological activity. In addition, obtaining these complexes under environmental and reproducible laboratory conditions is of critical importance. In this regard, this paper aims to develop an ecological, efficient and versatile synthetic method, able to produce A_3_B and A_4_ type metalloporphyrins, with a high degree of purity and a good reaction yield. Solvent free reactions activated by microwave irradiation have become increasingly important in the synthesis of mesoporphyrinic complexes due to their advantages such as shorter reaction times, higher reaction yields, absence of solvent in the reaction mixtures and increased selectivity of the synthetic reactions [15,16,17,18]. 

**Figure 1:**
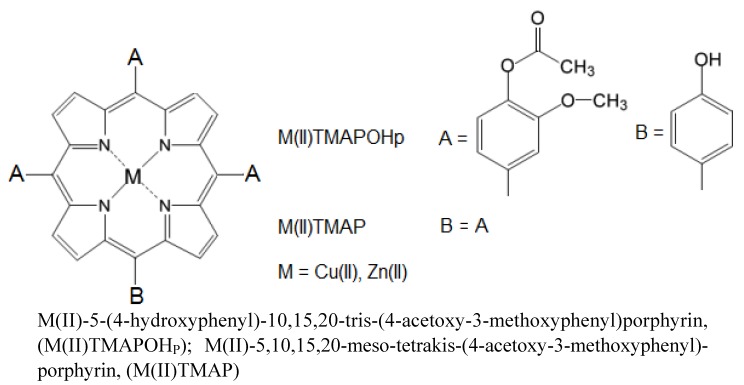
General structures of the mesoporphyriniccomplexes used in this study.

In this study synthesis of Zn(II)-5,10,15,20-*meso*-tetrakis-(4-acetoxy-3-methoxyphenyl) porphyrin, Zn(II)-5-(4-hydroxyphenyl)-10,15,20-tris-(4-acetoxy-3-methoxyphenyl) porphyrin, Cu(II)-5,10,15,20-meso-tetrakis-(4-acetoxy-3-methoxyphenyl) porphyrin and Cu(II)-5-(4-hydroxyphenyl)-10,15,20-tris-(4-acetoxy-3-methoxyphenyl) porphyrin (Figure 1), was carried out using microwave irradiation of the reaction mixtures consisting of substituted benzaldehydes, pyrrole and metallic salts in the presence of the basic catalyst in dry media. The reactions have been successfully repeated several times with identical results and then the mesoporphyrinic complexes were characterized by spectral analysis. In addition, a study of the solubility and their spectral behavior in environments with different polarities was performed.

## 2. Results and Discussion

Mesoporphyrinic complexes can be obtained by the classical pathway [19,20,21] that involves refluxing of equimolar ratios of the porphyrinic ligands and metal salts in the presence of a basic catalyst. This requires as a first step preparation of the porphyrinic ligand by condensation of pyrrole and substituted benzaldehydes in propionic acid at high temperature (~140 °C). Under these conditions, chlorins (reduced forms of the porphyrins) result from the synthesis as secondary products and isolation of mesoporphyrinic compound requires tedious chromatographic separations with decreasing reaction yield. 

In recent years, research specialists concerned with obtaining pharmacologically active mesoporphyrins have been focused on developing new ecological methods as an alternative method to the classical way in order to decrease the reaction times, improving the degree of purity of the final products and implicitly increasing the reaction yields [15,16,17,18,22,23,24,25,26,18,22]. 

Solvent-free synthesis by microwave irradiation has been successfully applied to obtaining mesoporphyrinic compounds because the absence of solvent from the reaction environment has the effect of decreased interaction time between reactant molecules and improves the reaction yield. Furthermore, specific conditions for initiating these synthetic reactions (absence of acidic medium, moderate temperatures) have eliminated the formation of chlorins and allow for an easy purification of the mesoporphyrinic compounds.

### 2.1. Infrared Spectra

The IR spectra recorded for the synthesized metalloporphyrins include typical vibration modes of both porphyrin macrocycle and phenyl substituents (Table 1) and infrared spectral assignments are generally in agreement with those previously reported for similar structures [27,28].

**Table 1:** Infrared spectral assignments of the mesoporphyrinic complexes (cm^−1^).

Assignments	Zn(II)TMAP	Cu(II)TMAP	Zn(II)TMAPOH_P_	Cu(II)TMAPOH_P_
**ν_O-H_**	-	-	3422 *m*	3420 *m*
**ν_C-H_**	2922 *m*	2923 *m*	2922 *m*	2924 *m*
**ν_C-H__from -O-CH3_**	2853 *m*	2852 *m*	2852 *m*	2853 *m*
**ν_C=O_**	1762 *m*	1763 *m*	1764 *m*	1765 *m*
**ν_C-N_**	1598 *m*	1600 *m*	1597 *m*	1598 *m*
**ν_C=N_**	1505 *m*	1501 *m*	1506 *m*	1503 *m*
**ν_C-Hpyrrole_**	1462 *m*	1461 *m*	1462 *m*	1462 *m*
**ν_C-O_**	1194 *s*	1196 *s*	1195 *s*	1196 *s*
**δ_C-H_**	998 *m*	1001 *m*	999 *m*	1000 *m*
**γ_C-C_**	860 *w*	862 *w*	868 *w*	858 *w*
**γ_C-N__pyrrole_**	797 *m*	800 *m*	798 *m*	788 *m*

The intensities of the signals are described as weak *(w)*, medium *(m)*, strong *(s)*.

The presence of the –OH functional group in the structure of the Zn(II)TMAPOH_P_ Cu(II)TMAPOH_P_ is confirmed by appearance in the IR spectra of a large band at about 3,420 cm^−1^ assigned to O-H stretching vibration. The medium band observed at ~2,923 cm^−1^ can be assigned to C–H vibrations of the phenyl groups. Also, in the IR spectra of the complexes, a medium band was evidenced at ~2,852 cm^−1^, corresponding to C-H vibration frequencies of the –O–CH_3_ group. 

The IR band identified in the spectral range of 1,762–1,765 cm^−1^ are assigned to C=O stretching vibration while the strong absorption band at about 1,196 cm^−1^ can be attributed to the C-O bond vibrations. The medium bands corresponding the stretching vibration C=N and C-N were highlighted in the spectral range of 1,500–1,505 cm^−1^ and 1,600 cm^−1^, respectively. Another band observed at ~1,462 cm^−1^ in the infrared spectra of the synthesized complexes is due to the stretching vibration of C-H bond motion of the porphyrin ring.

### 2.2. Absorption and Fluorescence Spectra

The analysis of mesoporphyrinic complexes by UV–Vis spectroscopy is an efficient method used to confirm their structure because their molecular absorption spectra are typical. These contain a intense Soret band (400–440 nm) as a result of the a_1u_ (π) → e_g_ (π∗) transition and less intense Q bands (500–650 nm) corresponding to the a_2u_ (π) → e_g_ (π∗) transitions [29].

**Figure 2:**
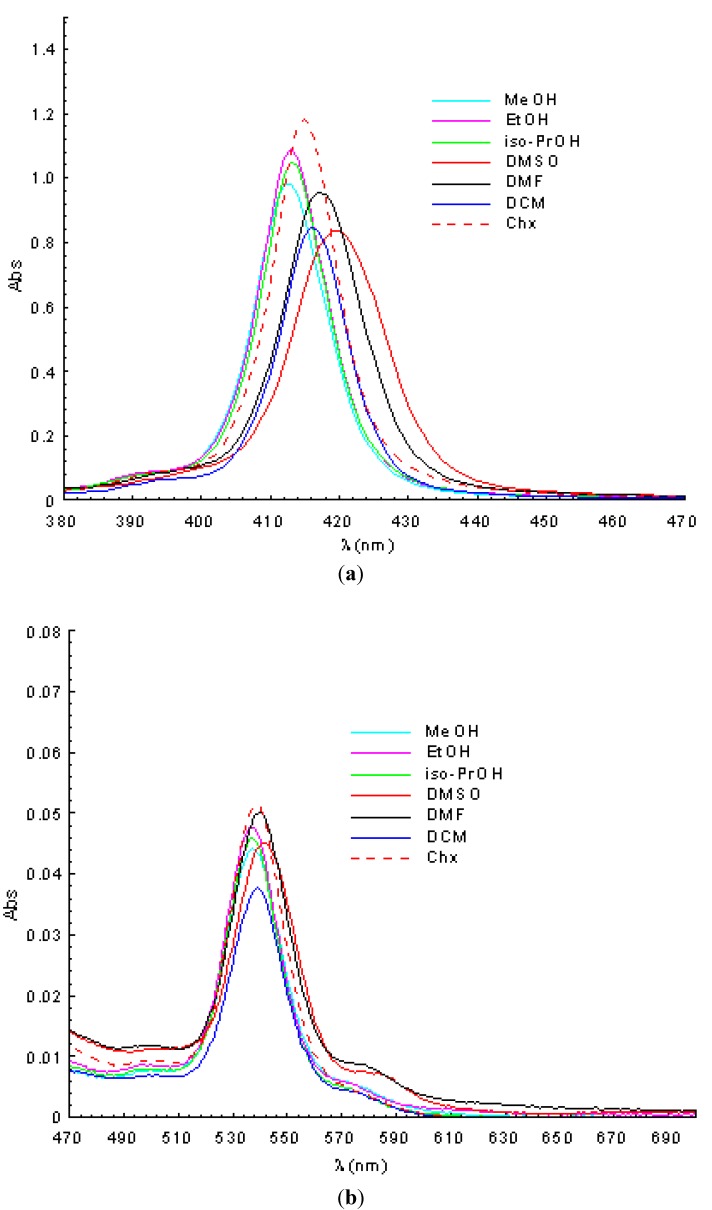
Absorption spectra of Cu(II)-5,10,15,20-*meso*-tetrakis-(4-acetoxy-3-methoxy-phenyl) porphyrin in different solvents (c = 2.5 × 10^−6^ M, a-Soret band; b-Q band).

UV-Vis spectral analysis reveals for the mesoporphyrinic complexes synthesized in this study a Soret band with a maximum in the range 412–430 nm, accompanied by Q bands situated in the 537–602 nm range (Figure 2 and Table 2). 

The analysis of the spectral data obtained for the zinc and copper mesoporphyrinic complexes in solvents with different polarities (Table 2) show that the main differences that occur between the absorption characteristics are determined by the nature of the metallic ion and environmental polarity. Thus, molecular absorption spectra of the Zn(II)TMAP and Zn(II)TMAPOHP displayed one Soret band (418–432 nm) and two Q bands (546–602 nm), while for both copper complexes Soret band (412–421nm) is accompanied by only one Q band (537–543 nm). 

As can be seen in Table 2, the Soret band of the copper porphyrins is hypsochromically shifted (~10 nm) compared to the zinc porphyrins having the same ligand. The Q bands of the copper porphyrins are blue shifted about 20 nm compared to the corresponding zinc porphyrins. These spectral shifts of the Soret and Q bands are the result of stronger conjugation effects that occur between the metallic ion orbital and the π electrons of the porphyrinic ring. For copper porphyrins these conjugation effects cause an energy decrease of the a_1u_ (π) and a_2u_ (π) orbitals relative to the e_g_ (π∗) orbitals, with increased energy available for electron transitions leading to a blue shift of the spectral bands compared to the zinc porphyrins [30]. 

The analysis of UV-Vis spectral data obtained for the porphyrinic complexes synthesized in this paper reveals changes in the relative positions of the Soret band and the Q bands, depending on the environmental polarity (Table 2). For the same porphyrinic complex, a small blue shift of the spectral bands was observed with increasing solvent polarity. These spectral changes can be associated of the physical interaction between the solvent molecules and the functional groups in the *meso* positions of the porphyrinic macrocycle.

**Table 2:** Maximum wavelength and molar absorptivity of the mesoporphyrinic complexes in different solvents (c = 2.5 × 10^−6^ M).

Solvent	λmax (nm) [lgε (L mol^−1^ cm^−1^)]				
	Soret band B(0,0)	Q bands Q_y_(0,0) Q_x_(1,0)			
***Zn(II)-*** ***5,10,15,20-meso-tetrakis-*** ***(4-acetoxy-3-methoxyphenyl) porphyrin***					
MeOH	422.5 [5.750]	556.8 [4.342]			595.8 [4.017]
EtOH	423.9 [5.769]	557.1 [4.318]			597.0 [3.924]
iso-PrOH	424.4 [5.750]	557.4 [4.292]			597.0 [3.857]
CH_2_Cl_2_	420.7 [5.718]	548.2 [4.342]			585.9 [3.681]
DMF	427.1 [5.744]	559.2 [4.326]			599.7 [3.833]
DMSO	429.6 [5.756]	561.0 [4.334]			601.2 [4.049]
Chx	418.0 [5.733]	549.4 [4.292]			601.8 [3.964]
***Cu(II)-*** ***5, 10, 15, 20-meso-tetrakis-*** ***(4-acetoxy-3-methoxyphenyl) porphyrin***					
MeOH	412.6 [5.595]		537.4 [4.292]		
EtOH	413.1 [5.639]		537.3 [4.107]		
iso-PrOH	413.3 [5,623]		537.3 [4.265]		
CH_2_Cl_2_	416.2 [5.528]		539.4 [4.182]		
DMF	417.3 [5.584]		539.9 [4.301]		
DMSO	419.6 [5.525]		541.7 [4.255]		
Chx	414.9 [5.674]		538.7 [4.318]		
***Zn(II)-*** ***5-(4-hydroxyphenyl)-10,15,20–tris-*** *(* ***4-acetoxy-3-methoxyphenyl*** *)* *** porphyrin***					
MeOH	423.0 [5.706]			556.1 [4.236]	597.2 [3.806]
EtOH	424.5 [5.725]			557.4 [4.283]	597.5 [3.880]
iso-PrOH	424.8 [5.735]			557.6 [4.274]	597.5 [3.833]
CH_2_Cl_2_	421.4 [5.678]			548.5 [3.301]	587.4 [3.602]
DMF	427.7 [5.693]			559.8 [4.292]	600.3 [3.982]
DMSO	430.2 [5.648]			561.4 [4.225]	602.3 [3.924]
Chx	418.4 [5.674]			546.6 [4.334]	601.0 [3.84]
***Cu(II)-*** *5**-(4-hydroxyphenyl)-10,15,20–tris-**(* ***4-acetoxy-3-methoxyphenyl*** *)* *** porphyrin***					
MeOH	413.3 [5.491]			538.0 [4.204]	
EtOH	413.6 [5.515]			537.6 [4.182]	
iso-PrOH	414.0 [5,505]			537.9 [4.170]	
CH_2_Cl_2_	416.4 [5.483]			539.5 [4.134]	
DMF	418.1 [5.465]			540.3 [4.215]	
DMSO	420.8 [5.389]			542.5 [4.167]	
Chx	415.2 [5.534]			542.5 [4.158]	

MeOH = methanol, EtOH = ethanol, iso-PrOH = isopropyl alcohol, DMF = dimethylformamide, CH_2_Cl_2_= dichloromethane, DMSO = dimethyl sulfoxide, Chx = cyclohexane.

**Figure 3:**
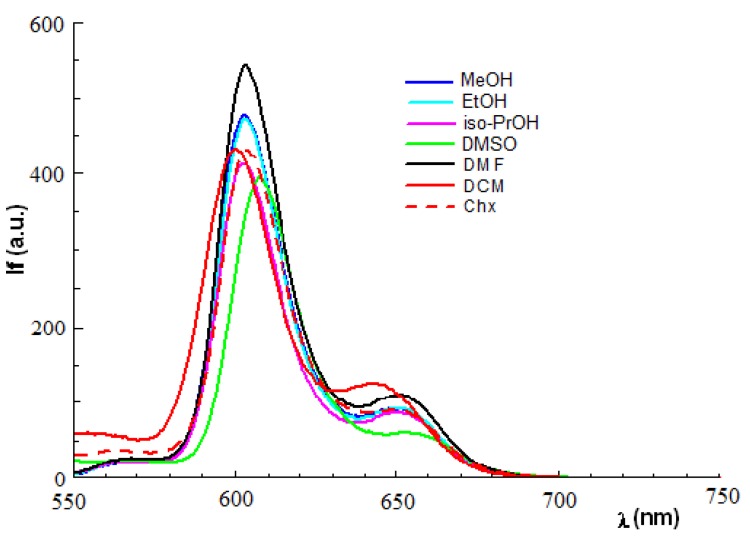
Fluorescence emission of Zn(II)-5-(4-hydroxyphenyl)-10,15,20-tris*-*(4-acetoxy-3-methoxyphenyl) porphyrin in different solvents (c = 2.5 × 10**^–^**^6^ M, λ_ex_ = 420 nm).

The fluorescence measurements were performed on the zinc mesoporphyrinic complexes in solvents with different polarities, at room temperature for λ_ex_ = 420 nm and their fluorescence spectral data are presented in Table 3. Figure 3 illustrates the fluorescence spectra of Zn(II)-5-(4-hydroxyphenyl)-10,15,20–*tris-*(4-acetoxy-3-methoxyphenyl)porphyrin in different solvents.

**Table 3:** The fluorescence data of zinc mesoporphyrinic complexes in different solvents(c = 2.5 × 10^−6^ M, λ_ex_ = 420 nm).

Solvent	^λ^max (nm) [I_f_] (a.u.)	
	Q(0,0)	Q(0,1)
***Zn(II)-*** ***5,10,15,20-meso-tetrakis-*** ***(4-acetoxy-3-methoxyphenyl)porphyrin***		
MeOH	602.1 [495.5]	650.0 [49.8]
EtOH	602.3 [534.2]	650.5 [112.7]
*iso*-PrOH	602.6 [537.8]	651.0 [116.2]
CH_2_Cl_2_	600.3 [476.7]	642.4 [139.7]
DMF	605.2 [533.1]	653.6 [89.2]
DMSO	607.0 [399.2]	656.5 [63.4]
Chx	605.8 [307.6]	653.5 [196.0]
***Zn(II)-*** ***5-(4-hydroxyphenyl)-10,15,20–tris-*** *(* ***4-acetoxy-3-methoxyphenyl*** *)* ***porphyrin***		
MeOH	602.3 [480.2]	650.0 [93.9]
EtOH	602.5 [472.0]	650.3 [93.9]
*iso*-PrOH	602.7 [413.3]	650.8 [86.9]
CH_2_Cl_2_	600.6 [432.5]	643.5 [128.0]
DMF	603.1 [544.8]	651.8 [112.7]
DMSO	607.6 [394.5]	654.7 [62.2]
Chx	603.9 [430.9]	651.0 [89.2]

MeOH = methanol, EtOH = ethanol, iso-PrOH = isopropyl alcohol, DMF = dimethylformamide, CH_2_Cl_2_= dichloromethane, DMSO = dimethyl sulfoxide, Chx = cyclohexane.

The fluorescence spectral data shows for Zn(II)TMAP and Zn(II)TMAPOH_P_ two emision bands located in the spectral region of 593–657 nm. 

Analyzing the influence of solvent polarity on the emission spectra of the two zinc porphyrins it was found that the shape of fluorescence spectrum does not depend on polarity of solvent under the experimental conditions used. The smaller blue shifts of the emission maxims by increase of environmental polarity reflect the physical interaction between the porphyrinic substituents and the solvent molecules. 

## 3. Experimental

### 3.1. General

Commercially available chemicals and solvents were used as received from Sigma-Aldrich and Merck. For the microwave assisted synthesis a CLATRONIC MWG775 H type temperature-controlled microwave oven was used. The elemental analysis of C, H and N was performed with an automatic Carlo Erba 1108 analyzer. IR spectra were recorded with a FT-IR 400D Nicolet Impact spectrophotometer. The substances under analysis, dried for 24 h at 150 °C, were processed as KBr pellets. The spectra were recorded in the 4,000–500 cm^−1^ spectral range. The NMR spectra of the zinc porphyrinic complexes were recorded with a 400 MHz Bruker NMR Spectrometer. EPR spectra of the copper porphyrinic complex were recorded on powders at room temperature using an ART-6 spectrometer, operating in the X band (9.01 GHz), equipped with a field modulation unit of 100 KHz. The UV-Vis spectra of the mesoporphyrinic complexes were recorded with the use of a Lambda 35 Perkin-Elmer spectrophotometer using a 10 mm path length quartz cell, in single beam mode. Fluorescence spectra were recorded with a Jasco FP 6500 spectrofluorimeter. The metalloporphyrin solutions in different solvents (methanol, ethanol, isopropyl alcohol, dimethylformamide, dichloromethane, dimethyl sulfoxide, cyclohexan) were freshly prepared in the spectrally pure solvents at the concentration 2.5 × 10^−6^ M and kept in dark until the measurement to prevent photodegradation. 

### 3.2. Synthesis of Zinc Mesoporphyrinic Complexes

A mixture of 4-hydroxybenzaldehyde (1.22 g, 0.01 mol), 4-acetoxy-3-methoxybenzaldehyde (5.82 g, 0.03 mol), pyrrole (2.76 mL, 0.04 mol), anhydrous zinc acetate (1.83 g, 0.01 mol) and 2–3 g of silica gel 60 (200–500 μm, 35–70 mesh) was subjected to microwave irradiation at 550 W for 8 min in the presence of 2,6-dimethylpyridine (0.1 mL). The presence of the metalloporphyrins in the reaction mixture was monitored by UV-Vis spectroscopy. Statistically, the reaction may provide a mixture of six metalloporphyrin isomers [A_4_, A_3_B, A_2_B_2_ (*cis* and *trans*), AB_3_ and B_4_-type]. Thin layer chromatography (dichloromethane/diethyl ether 30:1 v/v) has confirmed their presence in the final product while absence of chlorins was confirmed by monitoring the Q bands in the UV-Vis spectra.

The reaction product was extracted with dichloromethane/diethyl ether (30:1, v/v). The extract was filtered, the solvent was removed under vacuum and the product was purified by column chromatography, using silica gel (100–200 mesh size) as stationary phase and dichloromethane/diethyl ether (30:1, v/v) as eluent. In order of comparative evaluation the first two isomers were separated and characterized. The first band that passes through the chromatographic column corresponds to the Zn(II)-5,10,15,20-*meso*-tetrakis-(4-acetoxy-3-methoxyphenyl)porphyrin (A_4_ isomer) while the second band contains Zn(II)-5-(4-hydroxyphenyl)-10,15,20–tris-(4-acetoxy-3-methoxyphenyl)porphyrin (A_3_B isomer). The solutions of the zinc complexes were concentrated by simple distillation. The final zinc mesoporphyrinic complexes was obtained by preparative TLC (2 mm, silicagel 60 plates were used). 

Zn(II)-5-(4-hydroxyphenyl)-10,15,20–tris-(4-acetoxy-3-methoxyphenyl) porphyrin was obtained as violet crystals with a yield of 36%. Elemental analysis for C_53_H_40_N_4_O_10_ Zn: calculated C 66.45, H 4.18, N 5.85; found C 66.37, H 4.11, N 5.77. The chemical shifts of the NMR signals for the Zn(II)TMAPOH_P_ are as follows: ^1^H-NMR (CDCl_3_), δ_H_, ppm: 3.93 (s, 9H, *CH_3_COO*), 4.03 (s, 9H, *O-CH_3_*), 6.8 (s, 1H, *OH*), 7.25 (s, 3H, *H_ortho_^,^_-Ph-CH3COO_*), 7.42 (d, 3H, *H_ortho-Ph-CH3COO_*), 7.72 (d, 3H, *H_meta-Ph-CH3COO_*), 7.78 (d, 2H, *H_meta__-Ph-OH_*), 8.0 (d, 2H, *H_ortho-Ph-OH_*), 8.92 (d, 8H, *H_β__pyrr_*). ^13^C-NMR (CDCl_3_), δ_C_, ppm: 56.2, 76.7, 77.0, 115.8, 117.6, 119.1, 119.8, 126.2, 127.2, 128.8, 130.1, 132.3, 133.5, 146.8, 148.3, 149.6.

Zn(II)-5,10,15,20-*meso*-tetrakis-(4-acetoxy-3-methoxyphenyl) porphyrin was obtained with a yield of 23%. Elemental analysis for C_56_H_44_N_4_O_12_ Zn: calculated C 65.3, H 4.27, N 5.44; found C 65.22, H 4.18, N 5.33. The chemical shifts of the NMR signals for the Zn(II)TMAP are as follows: ^1^H-NMR (CDCl_3_), δ_H_, ppm: 3.75 (s, 12H, *CH_3_COO*), 3.96 (s, 12H, *O-CH_3_*), 7.03 (s, 4H, *H_ortho_^,^_-Ph-CH3COO_*), 7.40 (d, 4H, *H_ortho-Ph-CH3COO_*), 7.43 (d, 4H, *H_meta-Ph-CH3COO_*), 8.92 (d, 8H, *H_βpyrr_*). ^13^C-NMR (CDCl_3_), δ_C_, ppm: 56.2, 76.7, 77.3, 109.7, 115.8, 119.4, 128.6, 129.8, 134.5, 144.0, 145.2, 146.6. 

### 3.3. Synthesis of Copper Mesoporphyrinic Complexes

A mixture of 4-acetoxy-3-methoxybenzaldehyde (5.82 g, 0.03 mol), pyrrole (2.76 mL, 0.04 mol), 4-hydroxybenzaldehyde (1.22 g, 0.01 mol), anhydrous copper(II) chloride (1.34 g, 0.01 mol), 2–3 g of silica gel 60 (200–500 μm, 35–70 mesh) in the presence of 2,6-dimethylpyridine (0.1 mL) was irradiated in a Pyrex bottle by a microwave oven at 550 W for 8 min. The extent of the complexation reaction was monitored by UV-Vis spectroscopy. For this purpose, extraction of samples was performed after every 2 min of irradiation. Monitoring the reaction by UV-Vis and TLC revealed the presence in the final mixture of six isomers of porphyrin type. 

The first two copper mesoporphyrinic isomers (A_4_ and A_3_B type) were separated for this study. For this purpose the crude product was dissolved in dichloromethane/diethyl ether (30:1, v/v), filtered and finally purified on a chromatography column by repeated elution, using silica gel (100–200 mesh size) as stationary phase and dichloromethane/diethyl ether (30:1, v/v) as eluent. The first band that passes through the chromatographic column corresponds to the symmetrical copper porphyrin while the second band containing A_3_B isomer. The final copper mesoporphyrinic complexes was obtained by preparative TLC (2 mm, silicagel 60 plates were used). The solutions of the copper porphyrinic complexes were concentrated by simple distillation and the obtained dark red crystals were dried at ≈100 °C for 12 h. 

Cu(II)-5-(4-hydroxyphenyl)-10,15,20-tris-(4-acetoxy-3-methoxyphenyl)porphyrin was obtained with a yield of 38%. Elemental analysis for C_53_H_40_N_4_O_10_Cu: Calculated C 66.56, H 4.18, N 5.86; found C 66.45, H 4.12, N 5.78. 

Cu(II)-5,10,15,20-meso-*tetrakis*-(4-acetoxy-3-methoxyphenyl)porphyrin was obtained with a yield of 25%. Elemental analysis for C_56_H_44_N_4_O_12_Cu: Calculated C 65.4, H 4.28, N 5.45; found C 65.28, H 4.21, N 5.36.

EPR spectra of the copper mesoporphyrinic complexes were recorded in solid state at room temperature in order to obtain information about the coordination environment around copper ion. 

The EPR parameters evaluated for the copper porphyrinic complexes are: Cu(II)TMAP g_||_ = 2.165, g_⊥_ = 2.048, α^2^ = 0.7827, A_||_ = 202 × 10^−4^ cm^−1^ and g_||_ = 2.173, g_⊥_ = 2.05, α^2^ = 0.7942, A_||_ = 203 × 10^−4^ cm^−1^ for Cu(II)TMAPOH_P_. These magnetic parameters values are close to those reported in the literature for copper porphyrins and confirm a square planar geometrical arrangement of nitrogen atoms around the copper ion [31,32]. The α^2^ and g_||_ values indicates a covalent character of the copper-nitrogen bonds in the copper porphyrinic complex [33].

## 4. Conclusions

The main task of this study was to synthesize some A_3_B and A_4_ mesoporphyrinic type complexes by microwave irradiation under solvent-free conditions. The complexes were obtained in a short time with good yields (about 36% for the unsymmetrical mesoporphyrinic complexes), using an ecological method of synthesis. The structures of metalloporphyrins were confirmed by elemental analysis, UV-Vis, FT-IR, NMR and EPR spectroscopy. Furthermore the influence of the metallic ion and solvent polarity on spectral properties of the mesoporphyrinic complexes was investigated. The main differences observed in the spectral characteristics of the complexes are the result of the conjugation effects that occur between the central metallic ion orbital and the π electrons of the porphyrinic ring. The spectral changes that occur by increasing environmental polarity, which are smaller than those produced by the nature of the central ion, can be ascribed to the physical interaction between the solvent molecules and the functional groups in the *meso* positions of the porphyrinic macrocycles. 
